# Features of Cu and TiO_2_ in the flow of engine oil subject to thermal jump conditions

**DOI:** 10.1038/s41598-021-99045-x

**Published:** 2021-10-01

**Authors:** Sohail Ahmad, Kashif Ali, Kottakkaran Sooppy Nisar, Aftab Ahmed Faridi, Nargis Khan, Wasim Jamshed, T. M. Yunus Khan, C. Ahamed Saleel

**Affiliations:** 1 Centre for Advanced Studies in Pure and Applied Mathematics (CASPAM), Bahauddin Zakariya University, Multan, 60800 Pakistan; 2 Department of Basic Sciences and Humanities, Muhammad Nawaz Sharif University of Engineering and Technology, Multan, Pakistan; 3 Department of Mathematics, College of Arts and Sciences, Prince Sattam Bin Abdulaziz University, Wadi Aldawaser, 11991 Saudi Arabia; 4 Department of Mathematics, The Islamia University of Bahawalpur, Bahawalpur, 63100 Pakistan; 5 Department of Mathematics, Capital University of Science & Technology, Islamabad, Pakistan; 6 Research Center for Advanced Materials Science (RCAMS), King Khalid University, P.O. Box 9004, Abha, Asir 61413 Kingdom of Saudi Arabia; 7 Department of Mechanical Engineering, College of Engineering, King Khalid University, Abha, 61421 Kingdom of Saudi Arabia

**Keywords:** Mathematics and computing, Applied mathematics, Computational science

## Abstract

The recent work investigates the heat transfer attributes in the flow of engine oil which comprises of nano-particles such as Cu and TiO_2_. The performance of Copper and Titanium oxide is over looked in the flow of engine oil. The energy equation is amended by the features of thermal radiation, viscous dissipation, and heat generation. The mathematical model signifies the porosity, entropy generation and moving flat horizontal surface with the non-uniform stretching velocity. Quasi-linearization, which is a persuasive numerical technique to solve the complex coupled differential equations, is used to acquire the numerical solution of the problem. Flow and heat transfer aspects of Cu–TiO_2_ in the flow are examined against the preeminent parameters. The flow is significantly affected by the thermal jump conditions and porous media. It is observed here that the temperature as well as heat transport rate is reduced with the effect of involved preeminent parameters. However, such fluids must be used with caution in applications where a control on the heat transfer is required. We may conclude that the recent study will provide assistance in thermal cooling systems such as engine and generator cooling, nuclear system cooling, aircraft refrigeration system, and so forth.

## Introduction

The flow and heat transfer performance of single phase fluids appear to be very low because of the truncated values of thermal conductivity. Some sort of heat transferring fluids such as pure water, ethylene glycol, transformer oil, Graphene nanolubricant, and engine oil rather have better rates of thermal conductivities if their composition would amended. The demanding usages of such heat transferring fluids in the industries motivated the researchers to work on the improvement of thermal conductivity of these fluids with a view to reduce the cost and to save the energy consumptions. Mixing of solid substances into the single phase fluids was proposed to be one of the possible ways out to improve the thermolysis behavior of these fluids. With the passage of time, the experimental data revealed the fact that such type of unassertive mixing of solid particles to base fluid caused the sedimentation and precipitation to the flow field, which in turn, reduced the thermal conductivity rate. Masuda et al.^1^ analyzed the behavior of solid particles dispersion into the working fluid taking powdered form of Al_2_O_3_, SiO_2_ and TiO_2_ with pure water as a base fluid. They detected a notable escalation in the thermal conductivity but the precipitation of solid particles in the flow field has also been encountered during the analysis. The precipitation of particles causes the agglomeration into the flow passages which significantly reduces the flow velocity of the base fluid. Choi and Eastman^2^ proposed a novel class of engineered fluids [termed as nanofluids (NFs)] with suspended nano-particles in an effort to elevate the thermal conductivity of working fluids. The nano-meter sized metallic particles (size ranging 1–100 nm) enhanced the thermal conductivity by improving the heat transferal rate of fluids and reducing the collaging effects of flow lines (see Refs.^3,4^).

From the beginning of the twentieth century, the amalgamation of metallic particles and heat transferring fluids have revolutionized the industrial heating and cooling systems, petrochemical industries, paper and glass fabrication units, textile industry, food processing plants and heat exchanger systems. Nano**-**particles used in the working fluids are commonly metals, metallic oxides, nitrides, carbides, carbon and its different forms (e.g. Graphite, Diamond, Fullerene, Graphene, Nanotubes) while the frequently used base fluids are some organic liquids (such as ethylene glycol, propylene glycol, etc.), water, engine oil, bio-fluids, polymeric solution, lubricants and transformer oil. Many researchers explored the circumstances of the heat transfer improvement in nanofluids with different combinations of nanoparticles and base-fluids. Pak and Cho^5^ empirically complemented heat transfer analysis of water-alumina (Al_2_O_3_/H_2_O) and water-titania (TiO_2_/H_2_O) nanofluids with a number of increasing values of Reynolds number typically ranging from 10^4^ to 10^5^. They examined the increasing heat transfer trend in nanofluids in contrast with the base fluid (water). Xuan and Li^6,7^ considered Cu/transformer oil and Cu/water nanofluids and reported 60% enhancement of heat transfer through proposed nanofluids over against the base fluids. Some other examples of nanoparticles with different base fluid combinations taken by eminent researchers considering CNT can be perceived in the works of Wang et al.^8^ and Ding et al.^9^. Furthermore, Yoo et al.^10^ considered the thermal behavior of different nanofluids for TiO_2_, Al_2_O_3_ and Fe. Sajadi and Kazemi^11^ reflected the characteristics of TiO_2_ nanofluids, Ghazvini et al.^12^ taken into consideration the diamond-engine oil nanofluids, Ferrouillat et al.^13^ incorporated water–silica (SiO_2_/H_2_O) nanofluids. All these studies reveal that the rate of thermal conductivity goes on increasing with the addition of various nanoparticles to the base fluids.

The heat transfer performance of nanofluids mainly depends on thermal behavior of nano-sized suspended particles, their concentrations in base fluid, and mass flow rates of engineered fluids. Madhesh et al.^14^ studied through experimental observation; the rheological properties of Cu–TiO_2_/water nanofluid conceded a tube-shaped heat exchanger with an average size of 55 nm nanocomposites. They reported an enhancement of 49% and 52% in the values of local Nusselt number and convective heat transfer coefficient respectively. Hayat and Nadeem^15^ numerically scrutinized the heat transfer rate through a stretching surface with rotating frame of reference in the existence of heat generation, porosity and thermal radiation effects using Ag–Cu/water hybrid nanofluid. Batmunkh et al.^16^ also calculated the thermal conductivities of Ag–TiO_2_/water based nanofluid and concluded that Ag-particles can inevitably increase the thermal conductivity of base fluid embedded with TiO_2_. Further literature related to the field of composition and rheology of nanoparticles can be accessed by the references^17–20^.

The comparison of thermal conductivity of titanium dioxide (TiO_2_) and copper (Cu) nanoparticles with the engine oil (EO) as a base fluid is the main attention of current analysis because of its eminent features in the actuators, electronic devices, fuel cells, cooling systems, heat pumps and heat exchangers. TiO_2_ is a non-toxic, economical, stable ceramic material with relatively high thermal conductivity value (4.0–11.8 W m^–1^ K^–1^). Leong et al.^21^ experimentally analyzed the thermal behavior and heat source/sink aspects of a copper–titania (Cu–TiO_2_) hybrid nanofluid and matched with the characteristics of a conventional (Cu and TiO_2_) nanofluid concurrently. Ali et al.^22^ proposed a Brinkman-type fluid model to analyze the shape impacts of nanofluids considering engine oil and kerosene oil with MoS_2_ nanoparticles over a rotating surface. Vasheghani et al.^23^ analytically measured the viscosity and heat transferal rate of TiO_2_-engine oil nanofluid employing the hot-wire method and validated the results with experimental data. Recently, Khata et al.^24^ evaluated the thermal performance of water and engine oil nanofluids with different geometrical settings considering corrugated surfaces in the heat exchangers with larger values of Reynolds number for water and smaller values of Reynolds number for engine oil on the basis of experimental data. A comparative analysis of thermal behaviour of engine oil (EO) and ethylene glycol (EG) with gold (Au)-nanoparticles were presented by Rajo et al.^25^ considering time-dependent magnetic field and thermal radiation effects on the porous channel.

In the literature of the thermal behaviour of nanofluids with various nanoparticles for both Newtonian and non-Newtonian models through stretching surfaces are extensively available. Khan and Pop^26^ are pioneer researchers in considering the stretching surface for the flow of nanofluids. Ghadikolaei et al.^27^ presented a numerical comparison of the copper–water nanofluid and copper-titanium dioxide/water (Cu–TiO_2_/H_2_O) nanofluid over a stretching sheet along with different geometrical shapes of nanoparticles. They established the fact that the platelets shaped nanoparticles have superiority over the brick and cylinder shaped nanoparticles. In another study on the porous stretching surface, Ghadikolaei with Hosseinzadeh et al.^28^ inspected the thermal behaviour of titanium dioxide-ethylene glycol (Cu–TiO_2_/C_2_H_6_O_2_) nanofluid with convective boundary condition by employing RK shooting technique. Simulation analysis of Copper and Aluminum oxide hybrid nanofluid flow over a stretching surface immersed in a porous medium, under the influence of heat generation and induced magnetic field, was investigated by Ali et al.^29^. Ahmad et al.^30,31^ numerically examined the flow of gyrotactic microorganisms and nanofluids over a nonlinear stretching sheet.

In light of the aforementioned literature reviews, it is concluded that no work has been performed yet to numerically investigate the performance of nano-particles Cu and TiO_2_ in the flow of engine oil over a stretching surface subjected to thermal jump conditions. To the best of our knowledge and in the light of the preceding literature review, it is observed that the current analysis has not yet been explored before and is being reported for the first time in the literature. An incompressible fluid characterized by the relation of porous media is taken over an extending surface. Numerical solution is obtained by employing the Quasi-linearization technique. The impacts of numerous involved parameters on temperature and velocity profiles, Nusselt number, and skin friction coefficient have been analyzed through tabular and graphical data.

## Mathematical description of the problem

**Figure 1:**
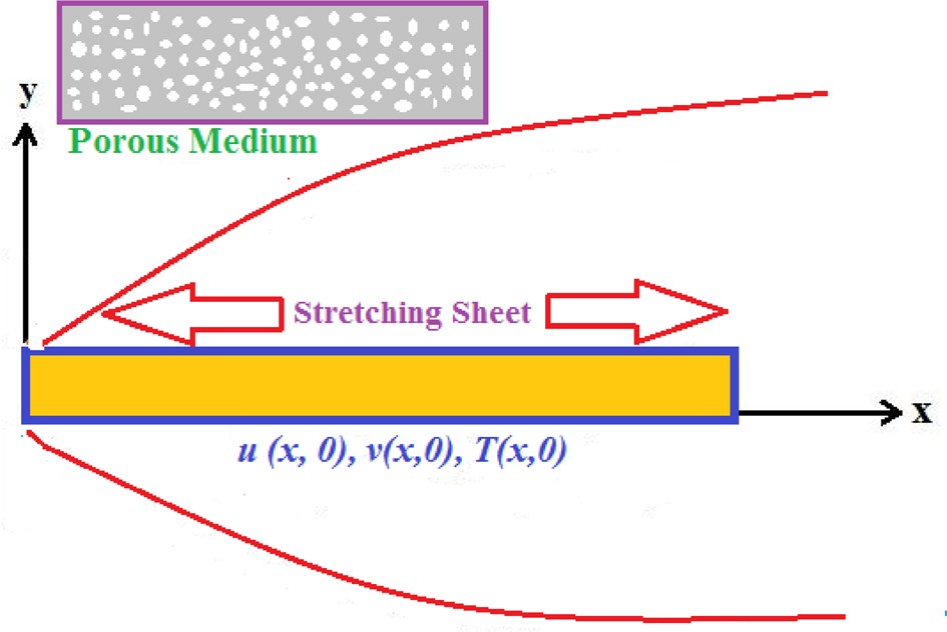
Flow sketch presentation.

We develop a mathematical model which represents the heat transfer analysis over a horizontal surface with the non-uniform stretching velocity $U_{w} (x,0) = bx$. A laminar incompressible and viscous flow of Oldroyd-B nanofluid is considered under usual boundary layer approximation through a porous media. The effects of variable thermal conductivity and radiative heat flux are also involved in the flow. Figure 1 represents the schematic diagram of the problem.

The governing flow equations are:

1 $$ \frac{\partial u}{{\partial x}} + \frac{\partial v}{{\partial x}} = 0, $$

2 $$ \begin{aligned} u\frac{\partial u}{{\partial x}} & + v\frac{\partial v}{{\partial x}} + \lambda_{1} \left[ {u^{2} \frac{{\partial^{2} u}}{{\partial x^{2} }} + v^{2} \frac{{\partial^{2} u}}{{\partial y^{2} }} + 2uv\frac{{\partial^{2} u}}{\partial x\partial y}} \right] = \frac{{\mu_{nf} }}{{\rho_{nf} }}\left[ {\left( {\frac{{\partial^{2} u}}{{\partial y^{2} }}} \right) - \frac{u}{k}} \right] \\ & + \frac{{\mu_{nf} }}{{\rho_{nf} }}\left[ {\lambda_{2} \left( {u\left( {\frac{{\partial^{3} u}}{{\partial x\partial y^{2} }}} \right) - \frac{\partial u}{{\partial x}}\left( {\frac{{\partial^{2} u}}{{\partial y^{2} }}} \right) + \frac{\partial u}{{\partial y}}\left( {\frac{{\partial^{2} u}}{{\partial y^{2} }}} \right) + v\left( {\frac{{\partial^{3} u}}{{\partial y^{3} }}} \right)} \right)} \right], \\ \end{aligned} $$

3 $$ \begin{aligned} u\frac{\partial T}{{\partial x}} & + v\frac{\partial T}{{\partial y}} = \frac{{k_{nf} }}{{\left( {\rho C_{p} } \right)_{nf} }}\left( {\frac{{\partial^{2} T}}{{\partial y^{2} }}} \right) - \frac{1}{{\left( {\rho C_{p} } \right)_{nf} }}\left( {\frac{{\partial q_{r} }}{\partial y}} \right) + \frac{1}{{\left( {\rho C_{p} } \right)_{nf} }}Q\left( {T - T_{\infty } } \right) \\ & + \frac{{\mu_{nf} }}{{\left( {\rho C_{p} } \right)_{nf} }}\left( {\frac{\partial u}{{\partial y}}} \right)^{2} . \\ \end{aligned} $$

The relevant boundary conditions of the problem are:

4 $$ \begin{aligned} & u(x,0) = U_{w} + \mu_{nf} \left( {\frac{\partial u}{{\partial y}}} \right),\,v(x,0) = V_{w} ,\, - k_{0} \left( {\frac{\partial T}{{\partial y}}} \right) = h_{f} (T_{w} - T), \\ & u \to 0,\,\frac{\partial u}{{\partial y}} \to 0,\,T \to T_{\infty } \,{\text{as}}\,y \to \infty . \\ \end{aligned} $$

### Thermal–physical characteristics of the Oldroyd-B nanofluid

The diffusion of nanometer-sized solid particles into base fluid causes an enhancement in the thermal–physical characteristics of the fluid under consideration. Table 1 summarizes the material parameters for the Oldroyd-B nanofluid composed by the accretion of copper and titanium oxide into the engine oil.

**Table 1:** Thermal–physical features of Oldroyd-B nanofluid.

Physical properties	Nano fluidics
Dynamic viscosity ($\mu$)	$\mu_{nf} = \mu_{f} (1 - \phi )^{ - 2.5}$
Density ($\rho$)	$\rho_{nf} = (1 - \phi )\rho_{f} + \phi \rho_{s}$
Heat capacity ($\rho C_{p}$)	$(\rho C_{p} )_{nf} = (1 - \phi )(\rho C_{p} )_{f} + \phi (\rho C_{p} )_{s}$
Thermal conductivity ($k$)	$\frac{{k_{nf} }}{{k_{f} }} = \left[ {\frac{{(k_{s} + (2)k_{f} ) - (2)\phi (k_{f} - k_{s} )}}{{(k_{s} + (2)k_{f} ) + \phi (k_{f} - k_{s} )}}} \right]$

The symbol $\phi$ represents the volumetric fraction coefficient of the nanoparticle and $\mu_{f}$ is the dynamic viscosity of the fluid. Where $\rho_{f} ,\,\,\,\,(C_{p} )_{f} \, \,{\text{and }}\,k_{f}$ characterize the density, specific heat capacity, and the thermal conductivity of the base fluid. Further, $\rho_{s} ,\,\,\,(C_{p} )_{s} \, \,{\text{and}}\, \, k_{s}$ indicate the density, specific heat capacity and the thermal conductivity of the nanoparticles. In the same way, $\rho_{nf} ,\,\,\,(C_{p} )_{nf} \, \,{\text{and}}\, \, k_{nf}$ denote the density, specific heat capacity and the thermal conductivity of the nanofluid.

The variable thermal conductivity is defined as:

5 $$ k_{nf} (T) = k_{nf} \left[ {1 + \varepsilon \frac{{T - T_{\infty } }}{{T_{w} - T_{\infty } }}} \right] $$

### Material properties of nanoparticles and base fluid

In the present work, heat transfer analysis through engine oil nanofluid has been accomplished taking into account the following material properties of the base fluid (engine oil) and the nanoparticles (copper and titanium oxide) as given in the Table 2.

**Table 2:** Material properties of base fluid and nanoparticles at 293 K.

Materials	$\rho ({\text{kg}}/{\text{m}}^{3} )$	$C_{p} ({\text{J}}/{\text{kgK}})$	$k({\text{W}}/{\text{mK}})$
Copper (Cu)	8933	385.00	401.00
Engine oil (EO)	884	1910	0.1440
Titanium oxide (Ti O_2_)	4250	686.20	8.9538

### Rosseland approximation

With the implication of the Rosseland diffusion approximation, the total radiative heat flux energy discharged through the heated sheet is proportionate to biquadratic order of its temperature that can be written as:

6 $$ q_{r} = - \frac{{4\sigma^{*} }}{{3k^{*} }}\frac{{\partial T^{4} }}{\partial y}. $$

here $\sigma^{ * }$ is the Stefan Boltzmann number and $k^{ * }$ is the absorption coefficient, where $T_{{}}^{4}$ is obtained by truncating higher-order terms of Taylor’s series expansion about $T_{\infty }^{{}}$.

7 $$ T^{4} \cong 4T_{\infty }^{3} T - 3T_{\infty }^{4} . $$

### Solution of the flow problem

The boundary value problem constructed in Eqs. (1–4) is transformed through similarity variables that facilitate us to convert the governing PDEs into corresponding ODEs. The stream function $\psi (x,y)$ associated with flow field is defined as:

8 $$ u = \frac{\partial \psi }{{\partial y}}, \, v = - \frac{\partial \psi }{{\partial x}}. $$

and the escorting similarity variables are of the form:

9 $$ \chi (x,y) = \sqrt {\frac{b}{{\nu_{f} }}} y, \, \psi (x,y) = \sqrt {\nu_{f} b} xf(\chi ), \, \theta (\chi ) = \frac{{T - T_{\infty } }}{{T_{w} - T_{\infty } }}. $$

Incorporating these similarity variables in Eqs. (1–4), we get:

10 $$ f^{\prime\prime\prime} + \phi_{1} \phi_{2} \left[ {ff^{\prime\prime} - f^{{\prime}{2}} + \beta 1(2ff^{\prime} - f^{2} f^{\prime\prime\prime}) + \beta_{2} (f^{{\prime\prime}{2}} - ff^{iv} ) - \frac{1}{{\phi_{1} }}Kf^{\prime}} \right] = 0, $$

11 $$ \theta^{\prime\prime}(1 + \frac{1}{{\phi_{4} }}\Pr Nr) + \Pr \frac{{\phi_{3} }}{\phi 4}\left[ {f\theta^{\prime} - f^{\prime}\theta + \theta \frac{Q}{{\phi_{3} }} + \frac{Ec}{{\phi_{1} \phi_{3} }}f^{{\prime\prime}{2}} } \right] = 0. $$

With recently transformed boundary conditions

12 $$ \left. \begin{gathered} f(0) = S, \, f^{\prime}(0) = 1 + \frac{\Lambda }{{\phi_{1} }}f^{\prime\prime}(0), \, \theta^{\prime}(0) = - B_{\varsigma } (1 - \theta (0)) \hfill \\ f^{\prime}(\chi ) \to 0, \, f^{\prime\prime}(\chi ) \to 0, \, \theta (\chi ) \to 0 \, \,{\text{ as }}\, \, \chi \to \infty \hfill \\ \end{gathered} \right\}, $$

where $\phi_{i} ^{\prime}s,{ 1} \le i \le 4$ involved in Eqs. (10–12) characterize the thermophysical properties of the engine-oil based nanofluid, and are defined as:

13 $$ \left. \begin{aligned} & \phi_{1} = (1 - \phi )^{2.5} , \, \phi_{2} = \left( {1 - \phi + \phi \frac{{\rho_{s} }}{{\rho_{f} }}} \right), \\ & \phi_{3} = \left( {1 - \phi + \phi \frac{{(\rho C_{p} )_{s} }}{{(\rho C_{p} )_{f} }}} \right),\phi_{4} = \left( {\frac{{(k_{s} + 2k_{f} ) - 2\phi (k_{f} - k_{s} )}}{{(k_{s} + 2k_{f} ) + \phi (k_{f} - 2k_{s} )}}} \right) \\ \end{aligned} \right\} $$

In this flow and heat transfer analysis, the main consideration to which physical quantity has been given is the local Nusselt number ($Nu_{x}$). It is equated in the following quotient form:

14 $$ Nu_{x} = \frac{{xq_{w} }}{{k_{f} (T_{w} - T_{\infty } )}}, $$

where $q_{w}$ represents the heat flux determined by

15 $$ q_{w} = - k_{nf} \left( {1 + \frac{16}{3}\frac{{\sigma^{ * } T_{\infty }^{3} }}{{k^{ * } \nu_{f} (\rho C_{p} )_{f} }}} \right)\left( {\frac{\partial T}{{\partial y}}} \right)_{y = 0} . $$

The non-dimensional transformation variables defined in (9), would yield

16 $$ Nu_{x} {\text{Re}}_{x}^{{ - \frac{1}{2}}} = - \frac{{k_{nf} }}{{k_{f} }}\left( {1 + Nr} \right)\theta^{\prime}(0), $$

where ${\text{Re}}_{x} = \frac{{U_{w} x}}{{\nu_{f} }}$ is the local Reynold number that mainly depends upon the stretching velocity ($U_{w} (x,0) = bx$ with $b > 0$, a stretching parameter).

### Entropy generation

Entropy generation has an indispensable role in all thermodynamic systems where the combined effects of mass and heat transfer are being analysed. The heat radiation, porosity and thermal conductivity cause various forms of irreversibilities in the flow and thermal gradients.

In the fore-stated flow model, the local entropy generation can be defined as:

17 $$ E_{G} = \frac{{k_{nf} }}{{T_{\infty }^{2} }}\left\{ {\left( {\frac{\partial T}{{\partial y}}} \right)^{2} + \frac{16}{3}\frac{{\sigma^{ * } T_{\infty }^{3} }}{{k^{ * } \nu_{f} (\rho C_{p} )_{f} }}\left( {\frac{\partial T}{{\partial y}}} \right)^{2} } \right\} + \frac{{\mu_{nf} }}{{T_{\infty } }}\left( {\frac{\partial u}{{\partial y}}} \right)^{2} + \frac{{\mu_{nf} u^{2} }}{{kT_{\infty } }}. $$

The dimensionless form of entropy generation rate is quantified as:

18 $$ N_{G} = \frac{{T_{\infty }^{2} b^{2} E_{G} }}{{k_{f} (T_{w} - T_{\infty } )^{2} }}. $$

Executing the similarity variables of Eq. (9), the dimensionless entropy generation rate can be put into the form:

19 $$ N_{G} = {\text{Re}} \left[ {\phi_{4} (1 + Nr)\theta^{{\prime}{2}} + \frac{1}{{\phi_{1} }}\frac{Br}{\Omega }(f^{{\prime\prime}{2}} + Kf^{{\prime}{2}} )} \right], $$

where, $Nr = \frac{{16T_{\infty }^{3} \sigma^{ * } }}{{3k_{f} k^{ * } }}$ is the radiation parameter and $B_{r} = \frac{{v_{f} U_{w} }}{{k_{f} (T_{w} - T_{\infty } )}}$ is the Brinkman number.

### Parameters of the problem

Following are the parameters involved in the dimensionless model equations:

**Table:** 

$\beta_{1}$	Deborah number	$\beta_{2}$	Deborah number
$K$	Porosity parameter	$S_{L}$	Slip parameter
$Ec$	Eckert number	$Q$	Heat generation parameter
$S$	Suction parameter	$\phi$	Nanoparticles volume fraction
$Nr$	Thermal radiation parameter	$B_{r}$	Brinkman Number
$Pr$	Prandtl number	$Re$	Reynolds number
$B_{i}$	Biot number	$\Omega$	Variable thermal conductivity parameter

## Methodology

We apply quasi-linearization method (QLM) to numerically solve the dimensionless transformed Eqs. (10) and (11). For this purpose, we formulate the sequences of the transformed functions $\left\{ {\hat{f}^{{(k)}} } \right\}\;{\text{and}}\;\left\{ {\hat{\theta }^{{(k)}} } \right\}$, which iteratively converge to the approximate solution of the dimensionless system. For details, see our earlier work^32–35^.

## Results and discussion

The prime concern of the present study is to numerically investigate the performance of nanoparticles such as Copper (Cu) and titanium oxide (TiO_2_) in the flow of engine oil over a stretching surface. The graphical and tabular representations of our findings, as well as their interpretations and discussion, are included in this section. The numerical calculations are determined by our developed code via the MATLAB. The effects of physical parameters against the dimensionless temperature, velocity, surface drag and local Nusselt number are computed numerically and analyzed through graphs and tables. We specify values of the parameters rather than assigning the particular values and the domain dimensions to the paramers^36,37^. The fixed values of the parameters in numerical calculations are: $\beta_{1} = 0.2$, $\beta_{2} = 0.2$, $K = 0.1$, $S_{L} = 0.05$, $Ec = 0.3$, $Q = 0.1$, $S = 0.05$, $\phi = 0.1$, $Nr = 0.1$, $B_{r} = 5$, $Pr = 6450$, $Re = 5$, $B_{i} = 0.1$, and $\Omega = 1$ otherwise specified.

**Figure 2:**
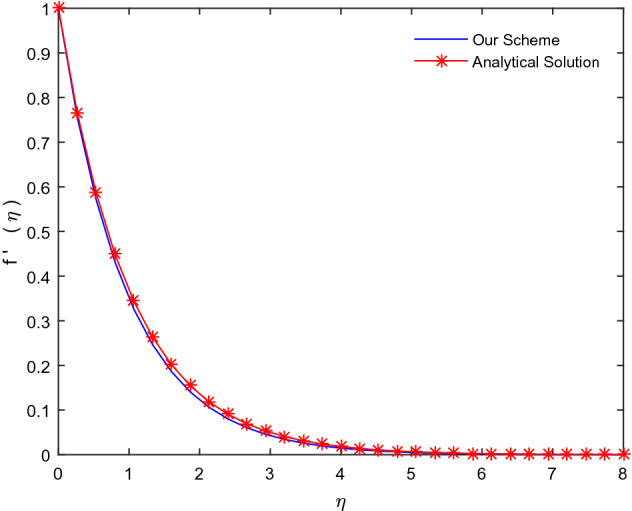
Comparison between numerical and analytical solutions.

In order to authenticate our numerical scheme, we compare our numerical results for the limiting case for the classical Newtonian fluid containing no additional nanostructures. In this case, the non-linear ordinary differential equations possess a simple analytical solution of the form $f(\eta ) = 1 - \exp ( - \eta )$. An excellent comparison in Fig. 2 validates our numerical scheme.

**Figure 3:**
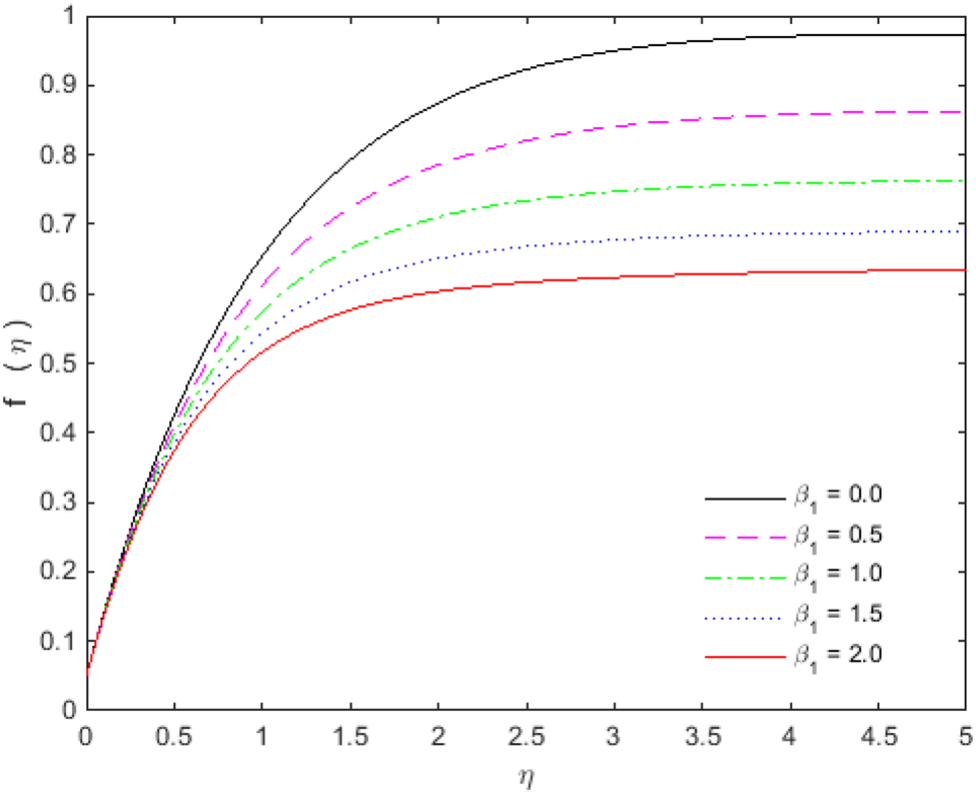
Normal velocity profile for $\beta_{1}$.

**Figure 4:**
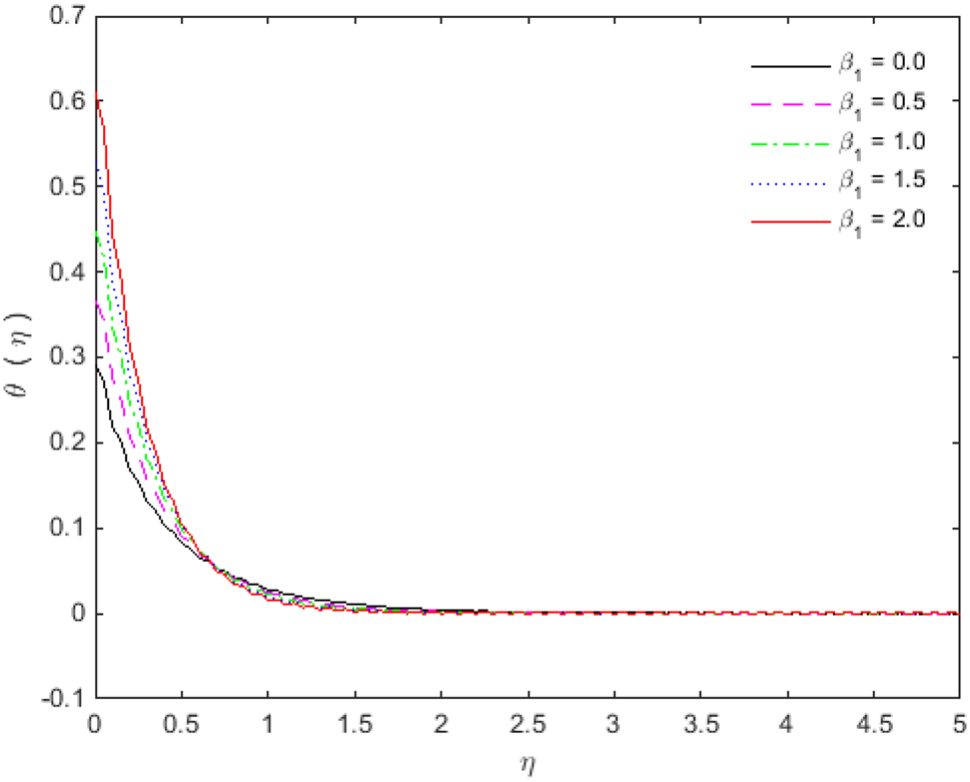
Temperature profile for $\beta_{1}$.

**Figure 5:**
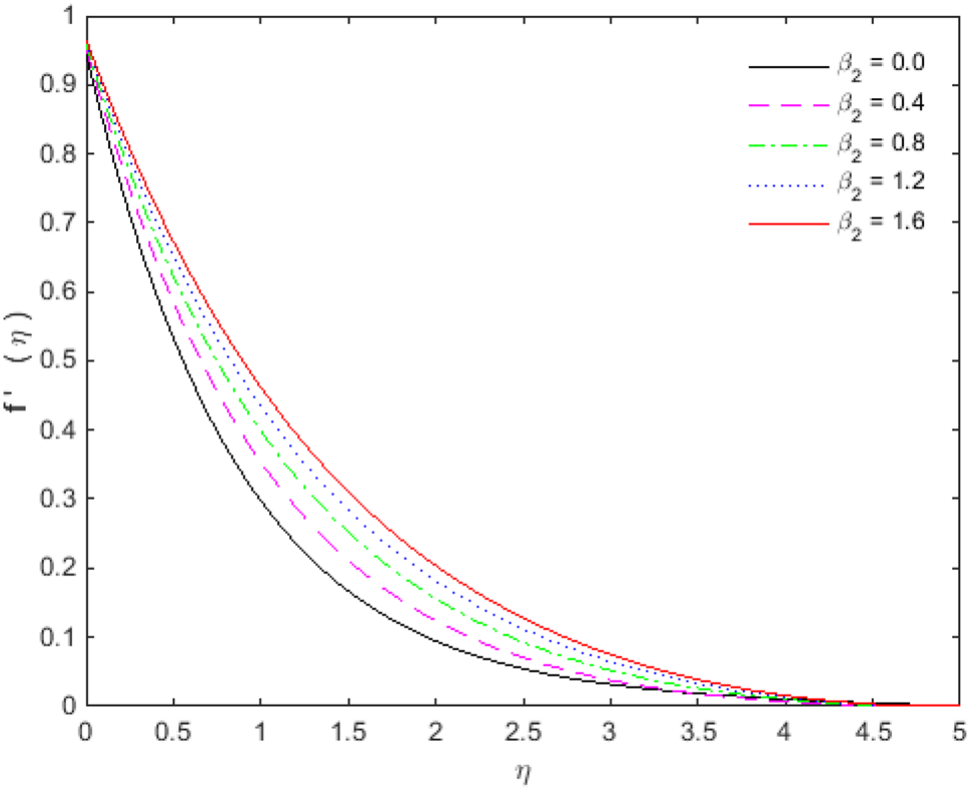
Streamwise velocity profile for $\beta_{2}$.

**Figure 6:**
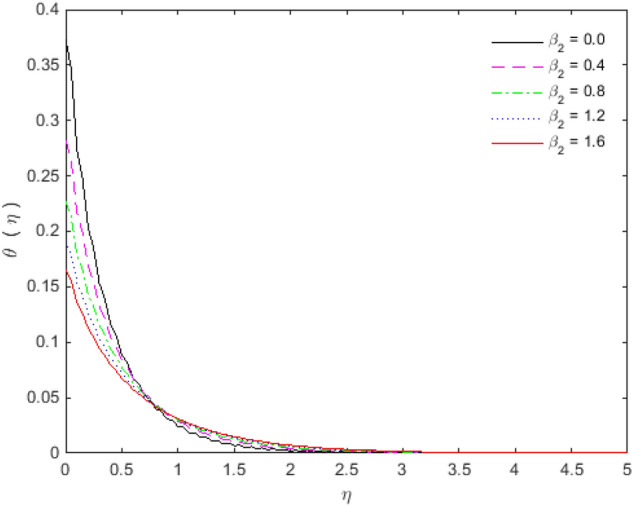
Temperature profile for $\beta_{2}$.

**Figure 7:**
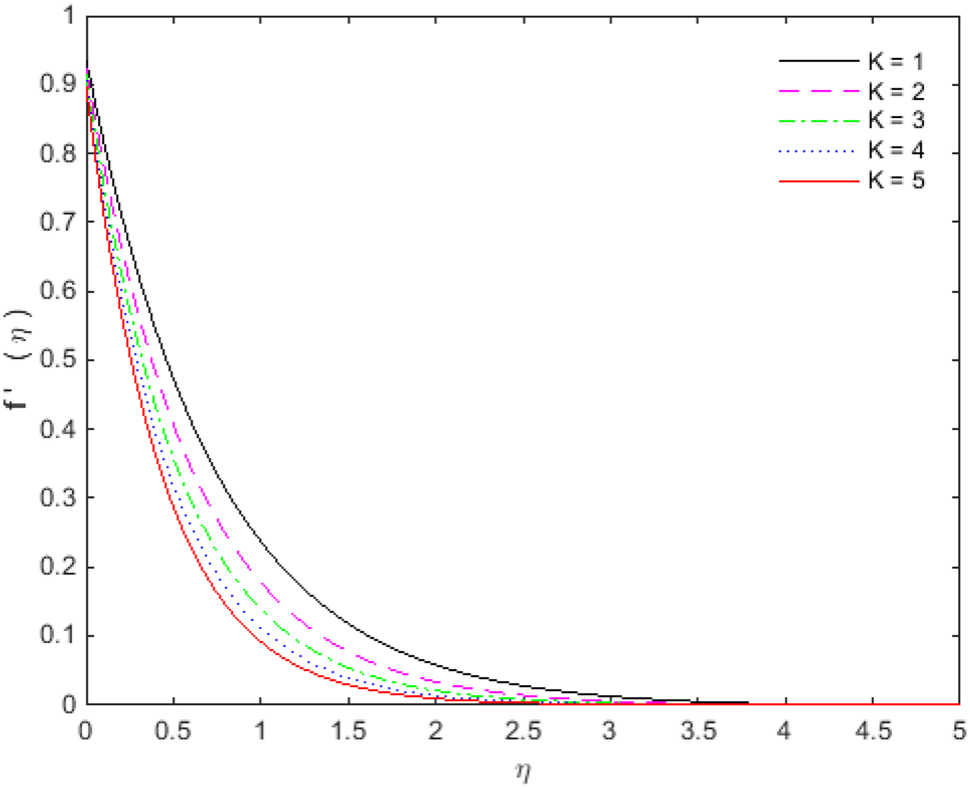
Streamwise velocity profile for $K$.

**Figure 8:**
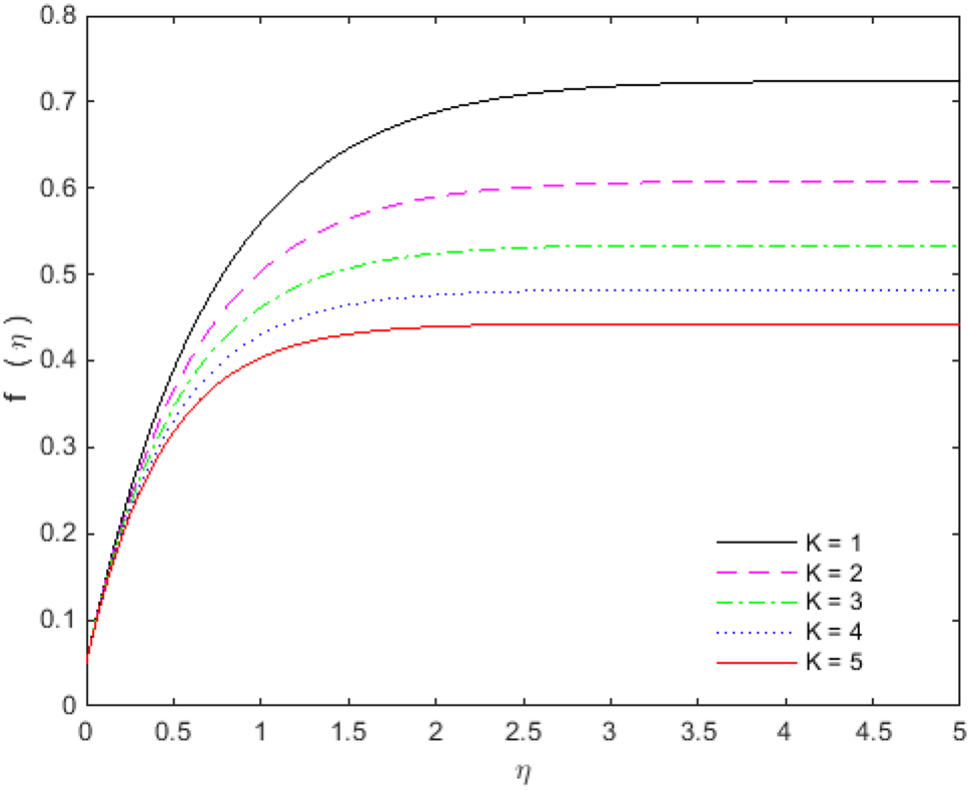
Normal velocity profile for $K$.

**Figure 9:**
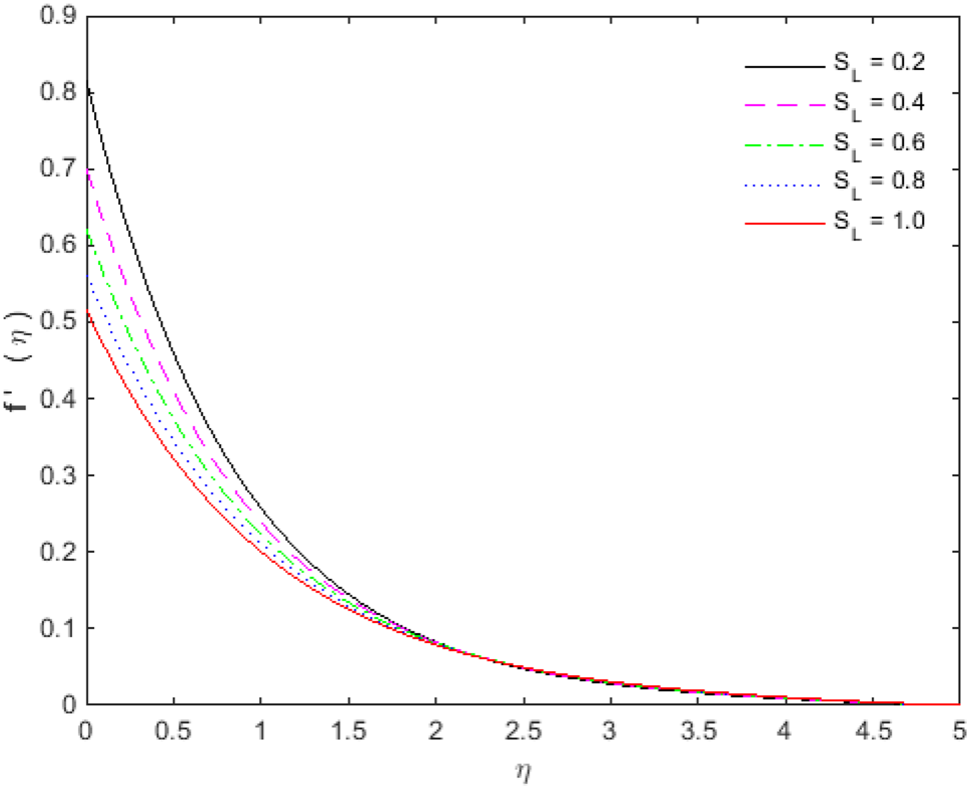
Streamwise velocity profile for $S_{L}$.

**Figure 10:**
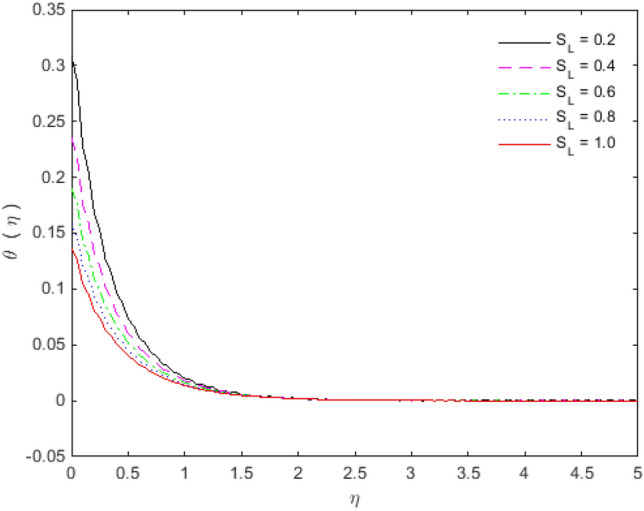
Temperature profile for $S_{L}$.

The appearances of velocities and temperatures for Deborah numbers $\beta_{1}$ and $\beta_{2}$ can be seen in Figs. 3, 4, 5 and 6. The effect of $\beta_{1}$ tends to accelerate the normal velocity but decelerate the temperature. On the other hand, the Deborah number $\beta_{2}$ produces opposite effects for velocities and temperature as compared to $\beta_{1}$. The dimensionless velocities (normal and streamwise) are escalated with the influence of porosity parameter (see Figs. 7 and 8). An increase in the momentum velocity layer thickness is observed which is associated with the large values of porosity while the fluid motion faces resistance for small porosity. Thus the utilization of permeable media is much eminent to control the flow nature and to maintain the heat transfer. The values of the physical parameters along with the sheet stretching velocity can be adjusted in such a way that we may attain certain factual applications of the concerned work. From the results of Figs. 9 and 10, it is found that the slip parameter $S_{L}$ tends to elevate the velocity and temperature profiles as well.

Table 3 is provided to analyze the effect of Deborah numbers $\beta_{1}$ and $\beta_{2}$ on shear stresses. The parameter $\beta_{1}$ tends to elevate the skin friction for both Copper and titanium oxide but $\beta_{2}$ devaluates the skin friction for both ${\text{Cu}}$ and ${\text{TiO}}_{2}$. The influence of porosity and slip parameter is to reduce the shear stress $f^{\prime\prime}(0)$ on sheet surface which can be observed from Table 4. However, if we increase the porosity then shear stress gets increase on the surface. This fact is attributed from the reason that large pores size does not interrupt the flow marginally and shear stress increases with its effect.

**Table 3:** Effect of $\beta_{1}$ and $\beta_{2}$ on shear stresses for ${\text{Cu}}$ and ${\text{TiO}}_{2}$.

$\beta_{1}$	$f^{\prime\prime}(0)$ for ${\text{Cu}}$	$f^{\prime\prime}(0)$ for ${\text{TiO}}_{2}$	$\beta_{2}$	$f^{\prime\prime}(0)$ for ${\text{Cu}}$	$f^{\prime\prime}(0)$ for ${\text{TiO}}_{2}$
0.0	− 1.08120	− 0.94162	0.0	− 1.23926	− 1.07305
0.5	− 1.21759	− 1.05152	0.5	− 1.05708	− 0.91808
1.0	− 1.34371	− 1.15732	1.0	− 0.94197	− 0.81447
1.5	− 1.46076	− 1.25580	1.5	− 0.85541	− 0.74021
2.0	− 1.57016	− 1.34801	2.0	− 0.78988	− 0.68367

**Table 4:** Effect of $K$ and $S_{L}$ on shear stresses for ${\text{Cu}}$ and ${\text{TiO}}_{2}$.

$K$	$f^{\prime\prime}(0)$ for ${\text{Cu}}$	$f^{\prime\prime}(0)$ for ${\text{TiO}}_{2}$	$S_{L}$	$f^{\prime\prime}(0)$ for ${\text{Cu}}$	$f^{\prime\prime}(0)$ for ${\text{TiO}}_{2}$
0.0	− 1.23482	− 1.05569	0.2	− 0.92408	− 0.82123
0.3	− 0.82487	− 0.74198	0.4	− 0.74650	− 0.67790
0.5	− 0.68279	− 0.62484	0.6	− 0.62984	− 0.58010
0.7	− 0.58504	− 0.54178	0.8	− 0.54659	− 0.50856
0.9	− 0.51319	− 0.47944	1.0	− 0.48387	− 0.45368

**Figure 11:**
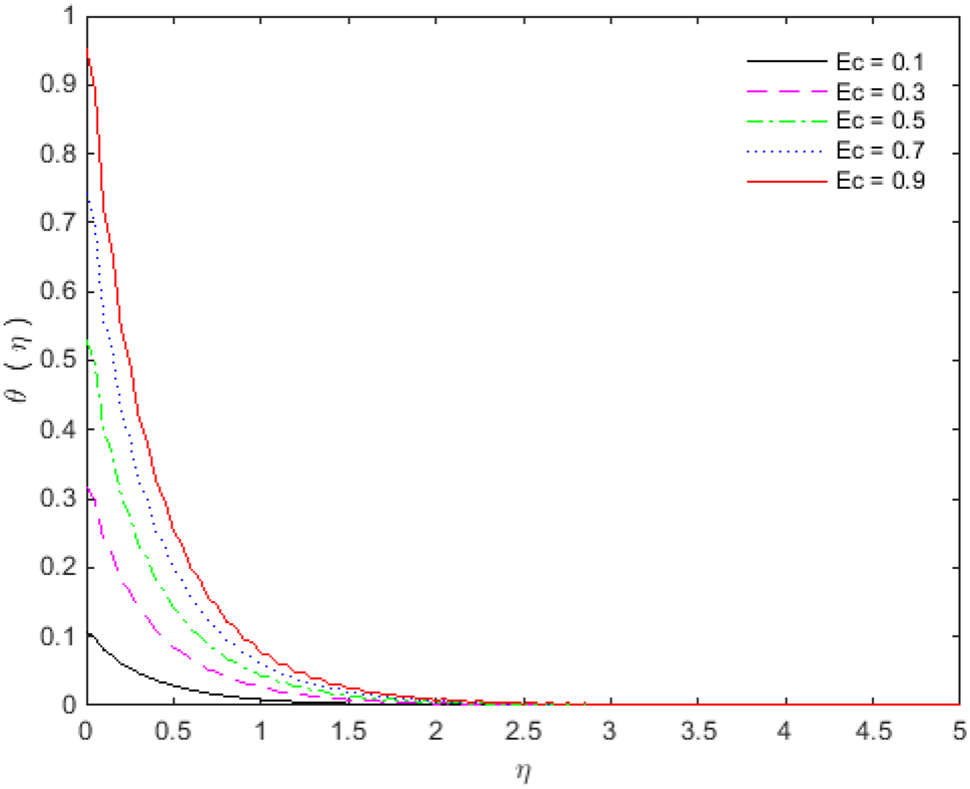
Temperature profile for $Ec$.

**Figure 12:**
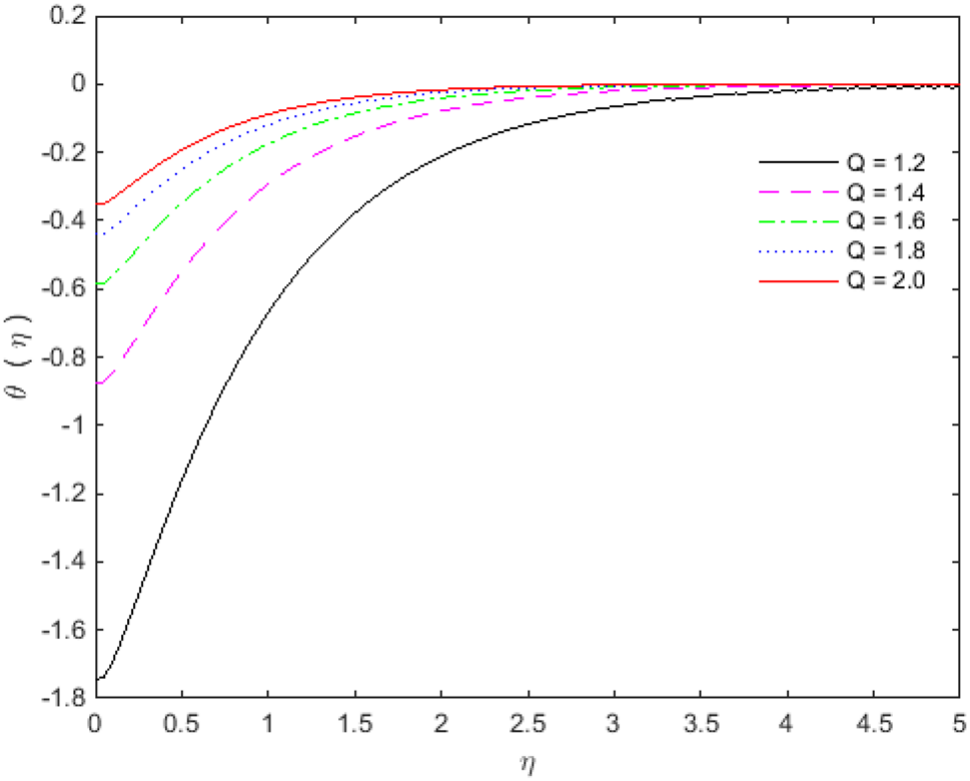
Temperature profile for $Q$.

**Figure 13:**
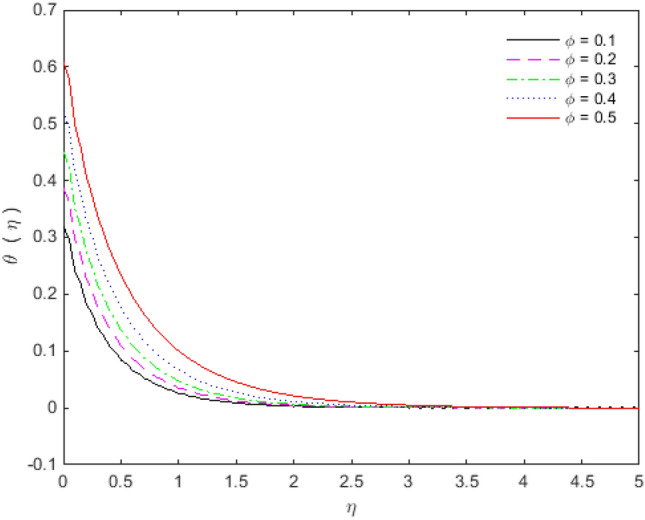
Temperature profile for $\phi$.

**Figure 14:**
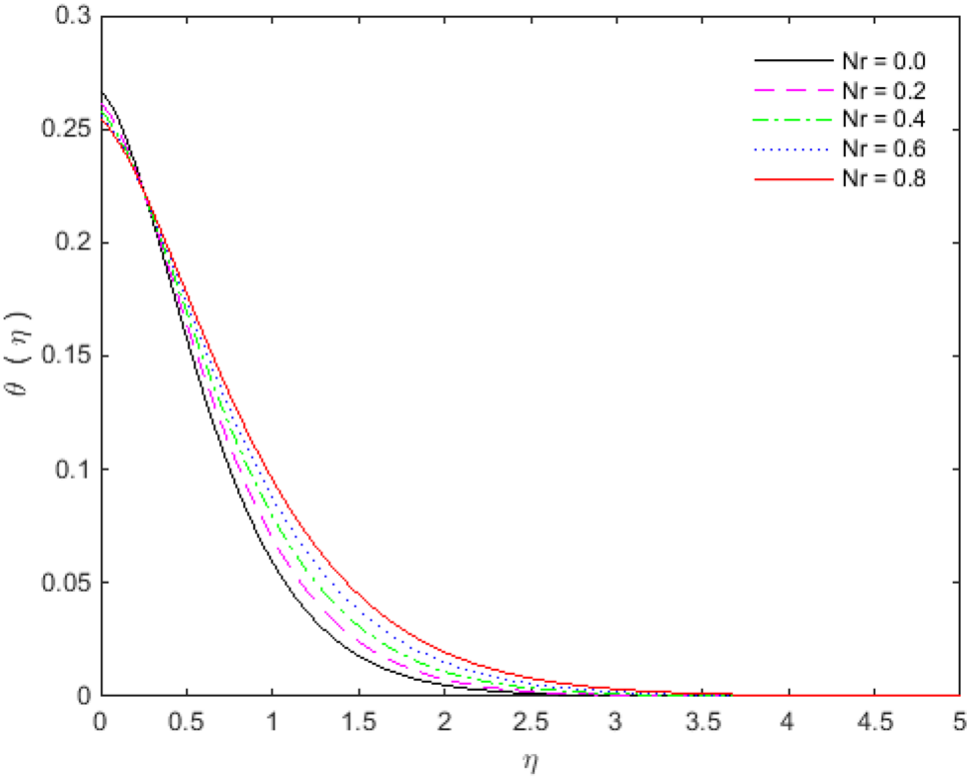
Temperature profile for $Nr$.

Figures 11, 12, 13 and 14 portray the impacts of Eckert number, heat generation, nanoparticles volume fraction, and thermal radiation on dimensionless temperature. Figure 11 portrays that an enhancement in the values of Eckert number tends to decelerate the temperature together with thermal boundary layer thickness. The ratio of the product of the difference between the temperatures of the fluid and the specific heat to the square of the fluid velocity refers to as the Eckert number for which $En = 0$ signifies no viscous dissipation. It is important to mention that fluid motion is desirably controlled by the Eckert number. A viscous force is generated under the action of viscous stress caused by the internal energy, so that; the viscous dissipation ($En < 0$ or $En > 0$) causes a decrease in the thermal boundary layer. Figure 12 is plotted to express the variation in temperature profile with the influence of heat generation parameter $Q$. The presence of $Q$ is responsible for decreasing thermal boundary layer thickness as the thermal state of the fluid is reduced by augmenting of the heat generation parameter $Q$.

**Figure 15:**
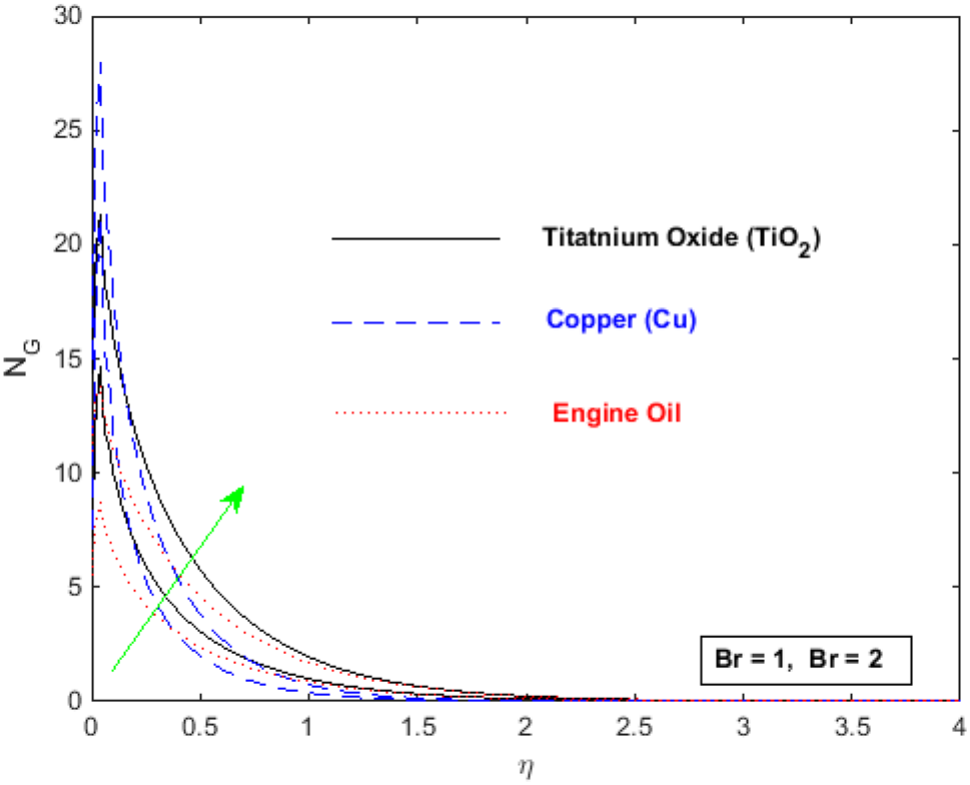
Entropy generation profile for $B_{r}$.

The effect of the nanoparticles volume fraction substantially reduces the temperature in the flow regime, as appeared in Fig. 13. Figure 14 elaborates the effects of radiation parameter $Rd$ over temperature distribution. It is observed that enhancement in radiation parameter $Rd$ causes a decrease in temperature. So, by augmenting the radiation parameter induces the decrease in fluid regime’s thermal diffusivity, which is responsible for reducing the heat energy in boundary layer. As a consequence, thermal boundary layer thickness and the fluid’s temperature are decreased. The values of $En$ and $Rd$ are taken in such a way that we may attain the optimal solution. The entropy generation $N_{G}$ increases for engine oil as well as for nanoparticles of titanium oxide and copper with an increase in Brinkman number $B_{r}$ (see Fig. 15). The velocity and temperature gradients can marginally affect the entropy generation. Because of the large temperature and velocity gradients, the entropy generation enhances at the sheet surface for *Cu, TiO*_*2*_ and engine oil.

The effect of Copper and Titanium oxide causes a decrease in the rate of heat transport over the surface of sheet for the Eckert number $Ec$ and the heat generation parameter $Q$ (see Table 5). A decrement in the values of heat transport rate for the change in solid nanoparticles volume fraction can be noticed from Table 6. The effect of thermal radiation is to upsurge the rate of heat transfer, as depicted in Table 6. The value of $Pr$ is taken as 6.135 in the analysis of thermal radiation. The parameter $Nr$ inserts low effect on $\theta^{\prime}(0)$ in the presence of viscous dissipation, porous medium and heat generation. The appropriate combination of nanoparticles volume fraction together with other parameters can provide the required heat transfer rate. The motion of the particles is in cross**-**over manner because of the existence of base fluid tends to move nanoparticles in cross**-**over manner due to which particles collision within the fluid produces more heat. On the other way, viscous dissipation also generates collision among the particles, and consequently heat transfer rate as well as fluid temperature decreases. The culminations portray that the shear stresses get reduced with Deborah number $\beta_{2}$, porosity $K$ and slip parameter $S_{L}$. In the same way, the heat transfer rates are diminished with the effect of nanoparticles volume fraction, viscous dissipation and heat generation. The present results are beneficial, with cautions, in applications where a control on the heat transfer is desired.

**Table 5:** Effect of $Ec$ and $Q$ on heat transfer rates for ${\text{Cu}}$ and ${\text{TiO}}_{2}$.

$Ec$	$\theta^{\prime}(0)$ for ${\text{Cu}}$	$\theta^{\prime}(0)$ for ${\text{TiO}}_{2}$	$Q$	$\theta^{\prime}(0)$ for ${\text{Cu}}$	$\theta^{\prime}(0)$ for ${\text{TiO}}_{2}$
0.1	− 0.08645	− 0.08924	1.2	− 0.35387	− 0.27447
0.3	− 0.05973	− 0.06809	1.4	− 0.22207	− 0.18764
0.5	− 0.03301	− 0.04694	1.6	− 0.18023	− 0.15846
0.7	− 0.00628	− 0.02579	1.8	− 0.15968	− 0.14380
0.9	0.02043	− 0.00464	2.0	− 0.14746	− 0.13498

**Table 6:** Effect of $\phi$ and $Nr$ on heat transfer rates for ${\text{Cu}}$ and ${\text{TiO}}_{2}$.

$\phi$	$\theta^{\prime}(0)$ for ${\text{Cu}}$	$\theta^{\prime}(0)$ for ${\text{TiO}}_{2}$	$Nr$	$\theta^{\prime}(0)$ for ${\text{Cu}}$	$\theta^{\prime}(0)$ for ${\text{TiO}}_{2}$
0.1	− 0.05973	− 0.06809	0.0	− 0.07330	− 0.07754
0.2	− 0.04703	− 0.06127	0.2	− 0.07383	− 0.07787
0.3	− 0.03529	− 0.05485	0.4	− 0.07417	− 0.07807
0.4	− 0.02356	− 0.04779	0.6	− 0.07439	− 0.07818
0.5	− 0.00949	− 0.03921	0.8	− 0.07451	− 0.07823

It has been come into noticed that the rate of heat transport are usually higher for resisting flows, but in the concerned problem, fluid flow is not substantially resisted by any internal or external source. The higher values of porosity are taken so that they may not influence the fluid flow. As the temperature of the fluid reduces for the heat generation, viscous dissipation and volume fraction of solid nanoparticles, so; this phenomenon will help to keep maintain the temperature of several engineering systems. The sum up results evidently discloses that use of Cu and TiO_2_ along with engine oil will cool down the heat up systems to a desirable extent. It is observed here that the temperature as well as heat transport rate is reduced with the effect of involved preeminent parameters. Therefore, we may conclude the recent study will provide assistance in thermal cooling systems.

## Conclusions

An inclusive numerical study of a fluid flow, involving Copper and Titanium dioxide with engine oil as base fluid over a moving flat horizontal stretching surface, is offered in this paper. The model involves several physical aspects of the problem. The flow also involves heat transfer attributes under the influences of viscous dissipation, heat generation and thermal radiation. A persuasive numerical technique named Quasi-linearization is utilized to attain the numerical solutions of the governing coupled flow model equations. The main culminations of this study may be listed as:

- The dimensionless velocities accelerate with the impact of porosity parameter.
- The parameters such as slip parameter and porosity parameter tend to devaluate the shear stresses on the sheet surface.
- The Deborah number $\beta_{1}$ causes an increase in the skin friction.
- The Eckert number, solid nanoparticles volume fraction and heat generation substantially decelerate the temperature of fluid and reduce the heat transfer rate as well.
- Both the velocity and temperature are enhanced by the slip parameter.
